# Pancreatic Steatosis: Innocent Bystander or Silent Culprit?

**DOI:** 10.5152/tjg.2026.26091

**Published:** 2026-07-20

**Authors:** Ahmet Sukru Alparslan, Ruhsen Ocal, Osman Cagin Buldukoglu, Serdar Akca, Berna Ozcanoglu, Mert Van, Galip Egemen Atar, Serkan Ocal, Ayhan Hilmi Cekin

**Affiliations:** 1 Department of Radiology, Antalya Training and Research Hospital, Antalya, Türkiye; 2 Department of Neurology, Antalya Training and Research Hospital, Antalya, Türkiye; 3 Department of Gastroenterology, Antalya Training and Research Hospital, Antalya, Türkiye

**Keywords:** Hepatosteatosis, migraine, pancreatosteatosis, white matter lesion, WMLs

## Abstract

**Background/Aims:**

Pancreatic steatosis (PS) is characterized by fat accumulation within the pancreas and is associated with chronic low-grade inflammation. Migraine, the third most commonly seen disease worldwide, is an established risk factor for white matter lesions (WMLs). Given the shared inflammatory background of these conditions, it was hypothesized that the presence of PS in patients with migraine may contribute to the development of WMLs.

**Materials and Methods:**

This cross-sectional study included patients with migraine who attended the outpatient clinic and had undergone cranial magnetic resonance imaging (MRI) scan for any indication within the last 6 months. Following MRI, patients were referred to the radiology department for transabdominal ultrasonography to assess the presence of PS and hepatosteatosis.

**Results: Among the:**

78 patients with migraine included in the study, 35 (44.9%) had cranial WMLs. PS was significantly more prevalent among patients with WMLs than among those without WMLs (57.4% vs. 16.7%, *P* < .001). In multivariable logistic regression analysis adjusted for age, body mass index, and low-density lipoprotein/high-density lipoprotein ratio, PS remained independently associated with cranial WMLs (odds ratio (OR) 5.79, 95% CI 1.004-33.41; *P* = .049). Increasing age was also independently associated with WMLs (OR 1.095 per year, 95% CI 1.018-1.179; *P* = .015).

**Conclusion:**

This study demonstrated that the presence of PS may contribute to disease burden in patients with migraine. Evaluation of PS in this population may help identify a potentially modifiable metabolic condition and provide additional insight into the management of this common and debilitating disorder.

**Main Points**

- Pancreatic steatosis (PS) and migraine are important clinical entities, which are driven by inflammation.
- PS is associated with an increase in white matter lesions (WMLs) in patients with migraine.
- Increasing age is independently associated with greater odds of cranial WMLs.

## Introduction

Pancreatic steatosis (PS), a condition associated with metabolic syndrome, has received increasing attention in recent years.^1^ Epidemiological studies have reported an estimated prevalence of up to 69%.^2^ Characterized by fat accumulation within the pancreas, PS has been associated with atherosclerosis, type 2 diabetes mellitus, acute and chronic pancreatitis, pancreatic cancer, and adverse surgical outcomes in patients with pancreatic disease.^3^

Migraine is a disabling neurological disorder with a complex pathophysiology.^4^ As the third most commonly seen disease worldwide, migraine is an established risk factor for white matter lesions (WMLs).^5^

WMLs are associated with gliosis, neuronal rarefaction, and axonal myelin loss, and a chronic proinflammatory state in patients with migraine has been proposed as an underlying factor in their development.^6^^-^^8^ Both PS and migraine have been associated with multiple inflammatory pathways.^9^^-^^11^ Given the shared inflammatory background of these disease conditions, it was hypothesized that the presence of PS in patients with migraine may contribute to the development of WMLs.

## Materials and Methods

### Study Design and Patient Population

This single center cross-sectional study was conducted prospectively between July 2025 and December 2025 at Antalya Training and Research Hospital. The study population consisted of patients with migraine evaluated in the outpatient neurology clinic. Patients with migraine were first screened to determine whether they had undergone cranial magnetic resonance imaging (MRI) scan for any indication within the last 6 months. Patients with had undergone MRI scan were then referred to the radiology department for transabdominal ultrasonography (TAU) to assess for PS and hepatosteatosis (HS). TAU evaluation was performed without knowledge of cranial MRI findings. Patients with migraine aged 18 years or above were included in the study. None were experiencing an acute migraine attack at the time of enrollment; however, subgroup analyses according to migraine subtype or disease-related characteristics such as the presence of aura or attack frequency were not performed. Patients with migraine who had diabetes mellitus, hypertension, cancer, or inflammatory-driven disorders (chronic infections as well as rheumatic diseases) were excluded because these conditions are major contributors to WMLs due to their proinflammatory effects and role in endothelial injury.^12^^,^^13^ Age and body mass index (BMI) of patients, low-density lipoprotein to high-density lipoprotein (LDL/HDL) ratio, triglyceride levels, glucose levels, smoking status, and the presence of PS and/or HS were recorded and analyzed.

This study was approved by the University of Health Sciences, Antalya Training and Research Hospital Ethics Committee (No: 2024-206, Date: July 11, 2024). Written informed consent was obtained from all participants. The study was conducted in accordance with the principles of Declaration of Helsinki. No AI assistance was utilized in this study.

### Ultrasonographic and Magnetic Resonance Imaging Evaluation

Ultrasonography was performed using a Toshiba Aplio 300 USG machine equipped with 4 MHz convex transducer probes. All ultrasound procedures were performed by a single radiologist with 15 years of experience, following standardized criteria to minimize interobserver variability. PS and HS were graded on a scale from 0 to 3. The grading systems described by Lee et al and by Needleman et al were used for the assessment of PS and HS, respectively.^14^^,^^15^

All MRI examinations in this study were performed using a 3 Tesla scanner (the Siemens Magnetom Skyra; Siemens Healthineers, Erlangen, Germany). The high magnetic field strength provided an increased signal-to-noise ratio, enabling high spatial resolution and optimal tissue contrast. A standardized brain MRI protocol was used for neurological evaluation, including axial T1-weighted imaging, axial and coronal T2-weighted imaging, axial fluid-attenuated inversion recovery sequences, diffusion-weighted imaging with corresponding apparent diffusion coefficient maps, susceptibility-weighted imaging, time-of-flight angiography, and gradient-echo–based fast field echo sequences. All images were acquired using a standard head coil with appropriate patient positioning and immobilization to minimize motion-related artifacts.

### Statistical Analysis

Statistical analyses were performed using IBM SPSS Statistics software version 27.0; (IBM SPSS Corp.; Armonk, NY, USA). Normality of continuous variables was assessed using the Shapiro–Wilk test. Normally distributed variables are presented as mean ± SD, whereas non-normally distributed variables are presented as median (range). Categorical variables are presented as counts and percentages.

Comparisons between patients with and without cranial magnetic resonance (MR) WMLs were performed using the independent samples *t*-test or Mann–Whitney *U*-test for continuous variables, depending on distribution, and the chi-square test for categorical variables.

To evaluate independent predictors of cranial WMLs, multivariable binary logistic regression analysis was performed. Variables associated with WMLs in univariate analyses at a threshold of *P* < .20, as well as clinically relevant factors, were included in the multivariable model. Based on these criteria, age, BMI, LDL/HDL cholesterol ratio, and PS were entered into the model.

PS was evaluated primarily as a dichotomous variable (presence vs. absence) to assess its overall association with cranial WMLs. Model results are presented as odds ratios (ORs) with 95% CIs. Model calibration was assessed using the Hosmer–Lemeshow goodness-of-fit test, and explanatory power was estimated using Nagelkerke *R*^2^.

All statistical tests were 2-sided, and a *P* value < .05 was considered statistically significant.

## Results

### Descriptive Characteristics

A total of 78 patients with migraine were included in the analysis, of whom 70 (89.7%) were female. The mean age of the study population was 43.7 ± 10.1 years (range, 19-63 years).

Cranial MRI demonstrated WMLs in 35 patients (44.9%), whereas 43 patients (55.1%) had no evidence of WMLs.

Baseline demographic and clinical characteristics according to the presence of cranial WMLs are presented in Table 1.

Patients with cranial WMLs were significantly older than those without WMLs (48.5 ± 9.6 vs. 39.8 ± 8.9 years, *P* < .001). BMI was higher in patients with WMLs (28.4 ± 5.8 vs. 25.9 ± 4.8 kg/m^2^), reaching borderline statistical significance (*P* = .049). The LDL/HDL cholesterol ratio was also significantly higher in patients with WMLs (*P* = .014).

PS was markedly more prevalent among patients with cranial WMLs than among those without WMLs (57.4% vs. 16.7%, *P* < .001). In contrast, HS, triglyceride levels, fasting glucose levels, and smoking status were not significantly associated with WMLs (all *P* > .20).

### Multivariable Logistic Regression Analyses

Variables with *P* < .20 in univariate analyses (age, BMI, LDL/HDL ratio, and PS) were included in the multivariable logistic regression model.

The overall model was statistically significant (omnibus *χ*^2^ = 24.94, *P* < .001). The model demonstrated good calibration, with a nonsignificant Hosmer–Lemeshow test (*P* = .693), and moderate explanatory power (Nagelkerke *R*^2^ = 0.420).

After adjustment for confounding variables, PS remained independently associated with cranial WMLs. Patients with PS had approximately 5.8-fold higher odds of WMLs compared with those without steatosis (OR 5.79, 95% CI 1.004-33.41; *P* = .049).

Increasing age was also independently associated with WMLs (OR 1.095 per year increase, 95% CI 1.018-1.179; *P* = .015).

BMI and the LDL/HDL cholesterol ratio were not statistically significant in the adjusted model (*P* = .173 and *P* = .110, respectively) (Table 2).

In summary, PS was significantly associated with the presence of cranial WMLs in patients with migraine. This association remained independent of age, BMI, and lipid profile in multivariable analysis. Age also emerged as an independent predictor of WMLs, whereas HS showed no association.

## Discussion

The aim of this study was to evaluate the association between PS and the presence of WMLs in patients with migraine. In this cohort, PS was independently associated with cranial WMLs, even after adjustment for age, BMI, and lipid profile. Age emerged as a robust and consistent predictor of cranial WMLs, whereas HS showed no association. Together, these findings suggest that PS may represent a potential extracranial marker associated with cerebral WMLs changes.

Pancreatic disorders encompass a wide variety of disease entities including acute and chronic pancreatitis, pancreatic exocrine insufficiency, PS, and pancreatic cancer.^16^^-^^18^ PS is defined as fat infiltration within the pancreas. The condition ranges from pancreatic inflammation to the development of pancreatic fibrosis and has been associated with etiological factors such as alcohol consumption, metabolic syndrome, infections, and toxins.^19^ Fat accumulation in the pancreas causes a low-grade chronic inflammation involving acinar cells and beta cells. This inflammatory state leads to endocrine and exocrine dysfunctions in the pancreas, as well as carcinogenesis.^20^ PS has also been reported to be prevalent in patients with cardiovascular disorders, carotid plaque, and inflammatory bowel disease.^21^^-^^23^

WMLs are radiological findings that may reflect gliosis, neuronal rarefaction, axonal loss, demyelination, and chronic microvascular injury. WMLs are relatively common findings which are triggered by insults to the central nervous system.^24^^,^^25^ In this cohort, cranial WMLs were present in 44.9% of patients with migraine, consistent with recent neuroimaging studies reporting WML prevalence of approximately 38%-45% among patients with migraine.^26^^-^^28^^,^^5^^,^^29^ Negm et al reported WMLs in 43.1% of patients with migraine, with lesion burden associated with migraine severity and aura.^30^ Dobrynina et al reported a WML prevalence of 38.7%-44.4% across migraine subtypes, with no WMHs observed in healthy controls.^27^ Al-Hashel et al similarly reported a high frequency of WML in migraine and identified several clinical risk factors including older age, frequent attacks, elevated serum homocysteine levels, and longer disease duration.^28^

PS was independently associated with approximately sixfold higher odds of cranial WMLs after adjustment for age, BMI, and LDL/HDL ratio. This represents the most notable and novel finding of the present study. Pancreatic fat accumulation, also referred to as fatty pancreas or nonalcoholic fatty pancreas disease, has been strongly associated with insulin resistance, type 2 diabetes mellitus, metabolic syndrome, and chronic low-grade systemic inflammation, highlighting the complex interplay between PS, HS, and endothelial dysfunction.^29^^-^^31^ A recent study by Chegini et al revealed that diet-induced inflammation is associated with increased odds of fatty pancreas, emphasizing its inflammatory nature.^3^ Salman et al demonstrated that reduction in pancreatic fat improves insulin resistance indices, underscoring the metabolic impact of PS.^32^ To the best of current knowledge, no prior study has specifically linked PS to cranial WMLs in migraine; the data therefore introduce pancreatic fat as a potential novel neurometabolic biomarker of structural brain injury in patients with migraine.

Age also emerged as a strong independent determinant of WMLs. This aligns with previous studies demonstrating age-dependent accumulation of WMLs in populations both with and without migraine.^28^^,^^5^^,^^33^ Age-related mechanisms include endothelial dysfunction, microvascular fragility, accumulated oxidative damage, and impaired glymphatic clearance, all of which may potentiate the effects of migraine-specific mechanisms such as cortical spreading depression and trigeminovascular activation.^34^^-^^37^ The data extend these observations by demonstrating that age remains a predictor even after adjusting for BMI, lipid profile, and PS, underscoring its central role in neurovascular vulnerability.

Pancreatic innervation comprises various complex pathways. The pancreas is innervated by the autonomic nervous system with sympathetic, parasympathetic, and sensory branches since early organogenesis.^38^ Two afferent pathways (spinal and vagal) and 2 efferent pathways (sympathetic and parasympathetic) form the pancreas–brain axis, with additional pathways to form the entero-pancreatic axis.^39^ Impairment of the pancreatic innervation has been shown to play a role in type 1 and type 2 diabetes and obesity.^40^ Future studies elucidating the crosstalk between the pancreas and brain may provide additional insight into the role of these organs in related disorders, potentially unraveling the underlying pathogenetic mechanisms of conditions such as PS and related clinical conditions.

The main strength of this study is the relationship revealed between PS and WMLs, whereas HS is not related to WML formation. This intriguing finding is the basis of this hypothesis-generating study, hopefully paving the path for future research on the topic. Inflammation is likely a shared pathogenetic mechanisms in both conditions, with PS-driven inflammatory state being a potential risk factor for WMLs. This finding is also important in terms of PS and its relationship with other inflammatory-driven disorders. Being a relatively new and hot topic, PS may represent a potential contributor to multiple disease states ranging from coronary heart disease, rheumatic disorders, and autoimmune disorders, owing to its inflammatory nature.

Several limitations should be acknowledged. First, the cross-sectional design precludes causal inference regarding the relationship between PS and WMLs. Second, the relatively small sample size may have limited statistical power and contributed to wide CIs. Third, migraine-specific clinical characteristics, including attack frequency, disease duration, aura status, and medication use, were not systematically recorded. In addition, inclusion of patients who had undergone cranial MRI for clinical indications may have introduced selection bias toward those with more severe neurological symptoms. Finally, inflammatory biomarkers and external control groups were not available. Therefore, these findings should be considered exploratory and hypothesis-generating and warrant confirmation in larger longitudinal studies.

In conclusion, this study suggests that PS may contribute to disease burden in patients with migraine. This hypothesis-generating study explored a previously uninvestigated potential association between PS and cranial WMLs in patients with migraine. Larger prospective studies including external control groups are needed to confirm and further refine these findings. Future studies evaluating the association of PS with both inflammatory-driven and atherosclerosis-related disorders may further clarify its role in disease pathogenesis. Assessment of PS in patients with migraine may provide additional insight into metabolic factors associated with cranial white matter changes. Larger prospective studies are required to confirm these findings.

## Figures and Tables

**Table 1.** Univariate Comparisons Between Patients with and Without Cranial WMLs

Variable	WMLs (n = 35)	No WMLs (n = 43)	*P*
Age, years (mean ± SD)	48.5 ± 9.6	39.8 ± 8.9	<.001*
BMI, kg/m^2^ (mean ± SD)	28.4 ± 5.8	25.9 ± 4.8	.049*
LDL/HDL ratio (median, range)	2.47 (1.08-5.22)	1.85 (0.66-3.50)	.014*
Triglyceride, mg/dL (median, range)	113 (66-362)	109 (28-381)	.764
Glucose, mg/dL (mean ± SD)	90.4 ± 13.2	88.0 ± 12.0	.371
Pancreatic steatosis, %	57.4	16.7	<.001*
Hepatic steatosis, %	68.8	55.8	.350
Smoking, %	37.1	25.6	.328

Data are presented as mean ± SD for normally distributed continuous variables, median (range) for non-normally distributed continuous variables, and percentage (%) for categorical variables.

BMI, body mass index; HDL, high-density lipoprotein; LDL, low-density lipoprotein; WML, white matter lesion.

*Level of significance: *P* < .05.

**Table 2.** Multivariable Logistic Regression Analysis for Predictors of Cranial WMLs

Variable	*B*	SE	OR (95% CI)	*P*
Age (per year increase)	0.091	0.037	1.095 (1.018-1.179)	.015*
LDL/HDL ratio	0.801	0.501	2.227 (0.834-5.947)	.110
Pancreatic steatosis (present vs. absent)	1.757	0.894	5.793 (1.004-33.410)	.049*
BMI (kg/m^2^)	0.088	0.064	1.092 (0.962-1.239)	.173

A multivariable binary logistic regression model was used to identify independent predictors of cranial WMLs. Results are presented as regression coefficients (*B*), SE, and OR with 95% CI. ORs represent the change in odds per 1 unit increase in continuous variables or for the presence vs. absence of categorical variables.

BMI, body mass index; HDL, high-density lipoprotein; LDL, low-density lipoprotein; OR, odds ratio; SE, standard error; WML, white matter lesion.

## Data Availability

The data that support the findings of this study are available on request from the corresponding author.
